# Predicting the occurrence of multidrug-resistant organism colonization or infection in ICU patients: development and validation of a novel multivariate prediction model

**DOI:** 10.1186/s13756-020-00726-5

**Published:** 2020-05-19

**Authors:** Li Wang, Xiaolong Huang, Jiating Zhou, Yajing Wang, Weizhang Zhong, Qing Yu, Weiping Wang, Zhiqiao Ye, Qiaoyan Lin, Xing Hong, Ping Zeng, Minwei Zhang

**Affiliations:** 1 Intensive Care Unit, Xiamen Hospital of Traditional Chinese Medicine, 1739 Xian Yue Road, Xiamen, 361009 Fujian Province China; 2 Intensive Care Unit, First Affiliated Hospital of Xiamen University, 55 Zhen Hai Road, Xiamen, 361000 Fujian Province China

**Keywords:** ICU, Multidrug-resistant organisms, Colonization, Infection, Pitt score, CRP

## Abstract

**Background:**

Multidrug-resistant organisms (MDROs) have emerged as an important cause of poor prognoses of patients in the intensive care unit (ICU). This study aimed to establish an easy-to-use nomogram for predicting the occurrence of MDRO colonization or infection in ICU patients.

**Methods:**

In this study, we developed a nomogram based on predictors in patients admitted to the ICU in the First Affiliated Hospital of Xiamen University from 2016 to 2018 using univariate and multivariate logistic regression analysis. We externally validated this nomogram in patients from another hospital over a similar period, and assessed its performance by calculating the area under the receiver operating characteristic (ROC) curve (AUC) and performing a decision curve analysis.

**Results:**

331 patients in the primary cohort and 181 patients in the validation cohort were included in the statistical analysis. Independent factors derived from the primary cohort to predict MDRO colonization or infection were male sex, higher C-reactive protein (CRP) levels and higher Pitt bacteremia scores (Pitt scores), which were all assembled in the nomogram. The nomogram yielded good discrimination with an AUC of 0.77 (95% CI 0.70–0.84), and the range of threshold probabilities of decision curves was approximately 30–95%.

**Conclusion:**

This easy-to-use nomogram is potentially useful for predicting the occurrence of MDRO colonization or infection in ICU patients.

## Introduction

Multidrug-resistant organisms (MDROs) have emerged as an important cause of morbidity and mortality in patients worldwide [1]. Escalating rates of antibiotic resistance add substantially to the morbidity, mortality, and costs related to infections in hospitalized patients, especially those in the intensive care unit (ICU) setting [2, 3]. However, progress in treatment has been limited. Few antibiotics have been developed in the last 30 years [4]. Fortunately, the relevant departments have formulated guidelines for the prevention and control of MDROs [5]. Among them, the rational use of antibiotics is an important item. Predictors for the occurrence of MDRO colonization or infection would also be valuable, aiding in empirical treatment when infection occurs.

Previously reported factors, such as recent (within 90 days) treatment with three or more antimicrobial agents, a previous MDRO colonization or infection, a hospital stay > 15 days, an indwelling catheter for more than 20 days, and long-term mechanical ventilation, were all strongly correlated with MDRO colonization or infection [6, 7]. However, some of these factors are based on patient recall of their history, and ICU patients often experience disturbances of consciousness, which introduce recall bias, while other factors display limited predictive value. Furthermore, single predictors may not be reliable in individual cases due to the complexity and heterogeneity of diseases in the ICU. Therefore, a model for predicting the occurrence of MDRO colonization or infection is desirable.

A nomogram is a statistical instrument that accounts for numerous variables to predict an outcome of interest for an individual patient [8]. Nomograms are routinely used to aid in decision making in cancer, trauma, neurocritical care, and other specialties [9–11]. The aim of this study was to construct an easy-to-use nomogram for predicting the occurrence of MDRO colonization or infection in ICU patients, with the aim of providing an aid for empirical treatment when infection occurs and for screening of high-risk patients for subsequent prospective randomized controlled studies.

## Materials and methods

### Study design and external validation

A retrospective observational study was conducted on a primary cohort of patients who presented to the ICU of the First Affiliated Hospital of Xiamen University between January 2016 and December 2018. We developed a nomogram from the primary cohort to predict MDRO colonization or infection in the ICU. For external validation, an independent cohort from another hospital, Xiamen Hospital of Traditional Chinese Medicine, was retrospectively enrolled over a similar period, using the same inclusion and exclusion criteria.

This study was approved by the Medical Ethics Committee of the First Affiliated Hospital of Xiamen University and the Medical Ethics Committee of Xiamen Hospital of Traditional Chinese Medicine. Informed consent was waived because the study was conducted retrospectively and no interventions were applied.

### Study population and definitions

Patients were eligible for inclusion if they were aged ≥18 years and underwent at least one microbial culture during their stay in the ICU. The following exclusion criteria were used: MDROs were detected before the patient entered the ICU or within the first 48 h in the ICU.

Qualified samples were collected of cerebrospinal fluid, sputum, urine, faeces, secretions, serous cavity effusion, bile, blood, catheters, etc. The multidrug-resistant status of the cultured bacteria was defined according to the provisional standard definition of MDROs published in Clinical Microbiology and Infection in 2012. MDROs can be defined as those organisms that are simultaneously resistant to three or more antimicrobial agents [12]. MDROs colonization was defined as the presence of MDROs cultured from microbiology specimens without evidence of tissue invasion or inflammation at that body site. MDROs infection was defined as the invasion of the body tissues by MDROs resulting in disease [13]. MDRO colonization or infection mentioned in this study refers to the occurrence during the ICU stay. The neutrophil-to-lymphocyte ratio (NLR) was determined by dividing the absolute neutrophil count (N) by the absolute lymphocyte count (L) [14]. The immunosuppressive status refers to acquired immune deficiency syndrome, advanced malignant tumors, transplantation, splenectomy, long-term oral immunosuppressant use, etc. Patients with MDRO colonization or infection constituted the MDRO group, and those without MDRO colonization or infection constituted the non-MDRO group.

### Data collection and measurements

Demographic and clinical data were collected from the included patients by research coordinators and board-certified ICU physicians using a case report form. They reviewed the electronic medical records and verified the final data. The following data, which may be related to MDRO colonization or infection according to the literature and clinical experience, were extracted: general patient characteristics, including age, gender and comorbidities; previous hospitalization history; bacteriological and drug resistance test results; mechanical ventilation status, the use of pressure increasing drugs, tracheal intubation / incision, indwelling urethral catheter or central venous catheter, state of consciousness and the use of cardiopulmonary resuscitation, within the first 24 h in the ICU; the levels of albumin (ALB), creatinine (Cr), total bilirubin (TBil), platelets (PLTs), oxygenation index (PaO_2_/FiO_2_), leucocyte count (WBC), N, neutrophil percentage (N%), L, lymphocyte percentage (L%), procalcitionin (PCT) and C-reactive protein (CRP), within the first 48 h in the ICU; etc.. Laboratory indicators were collected within the first 48 h in the ICU because some data was not available within the first 24 h, and then circulating neutrophils and lymphocytes were counted to calculate the NLR. To predict the occurrence of MDRO colonization or infection, the Sequential Organ Failure Assessment (SOFA) score, Acute Physiology and Chronic Health Evaluation (APACHE) II score and Pitt bacteremia score (Pitt score) were calculated at the baseline from the information available in the ICU registry at admission [15, 16]. Pitt score is a severity of illness grading system, evaluating mental status, presence or absence of fever, hypotension, mechanical ventilation and cardiac status [15].

### Statistical analysis

For the development of the nomogram, we searched for potential factors associated with MDRO colonization or infection reported in previous studies or based on clinical experience, that are able to be easily acquired in the early stages of ICU hospitalization. Randomly missing data were filled by using Monte Carlo multiple imputation methods [17]. Continuous data were reported as the medians [interquartile ranges]. Categorical data were presented as frequencies or ratios. A univariate logistic regression model was used to analyze factors that may be related to MDRO colonization or infection. Variables with a *P* value of 0.2 or less in the univariate logistic regression analyses were subjected to multivariate logistic regression analysis. The final selection of the multivariate logistic regression model was based on backward stepwise regression with the Akaike Information Criterion (AIC) [18]. A nomogram was formulated based on the results of the multivariate analysis of the primary cohort with the “rms” package, which allowed us to obtain occurrence probability estimates [19].

We tested the accuracy of the nomogram by determining its discrimination using an external validation cohort. The predictive ability of the nomogram was quantified by calculating the area under the receiver operating characteristic (ROC) curve (AUC). An AUC value of 1.0 indicates a perfect prediction, while 0.5 is equivalent to the toss of a coin. Furthermore, we used decision curve analysis to assess the benefits of nomogram-assisted decisions in a clinical context [20]. A decision curve analysis incorporates the clinical consequences of using a prediction rule by applying a different weight to true and false positives. This weighting is varied to reflect different patient preferences or differences in opinion about the risks of a procedure. These preferences are expressed in terms of a threshold probability for an action [21]. The present study was compliant with the standard guidelines for prediction models [22].

All analyses were performed using R software (version 3.4.2, http://www.r-project.org/). All statistical tests were two-sided, and *P* values of less than 0.05 were considered statistically significant.

## Results

### Patient characteristics

**Fig. 1:**
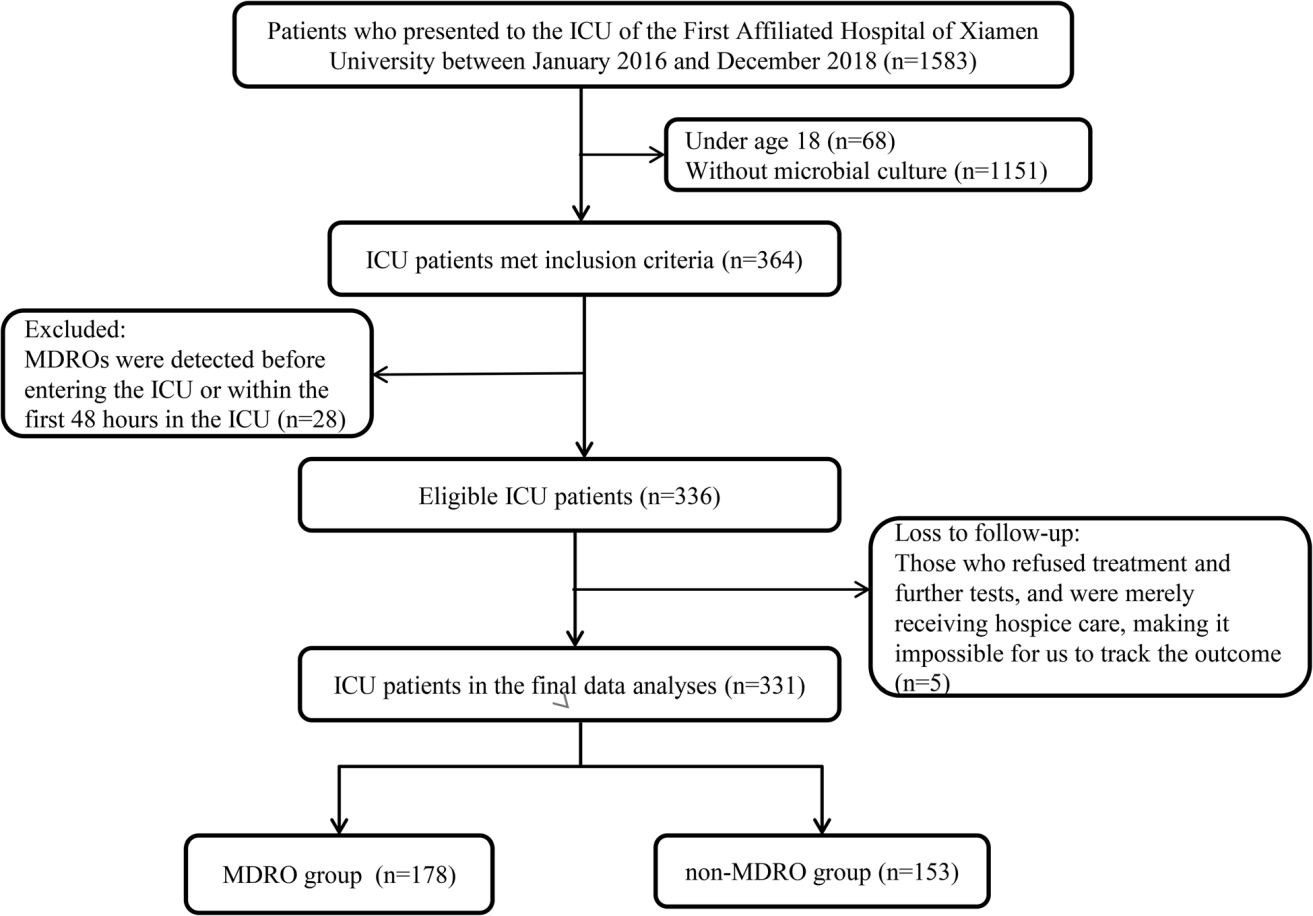
Flow chart for screening eligible patients

The flow chart of the process used to screen eligible patients is presented in Fig. 1. Of 364 patients who met the inclusion criteria, 336 patients were eligible. Among them, 5 patients were lost to follow-up. Finally, 331 patients in the primary cohort were included in the statistical analysis.

The main characteristics of the study population are shown in Table 1. In the primary cohort, 178 patients (53.8%) comprised the MDRO group, and 153 patients (46.2%) constituted the non-MDRO group. In the validation cohort, 97 patients (53.6%) comprised the MDRO group, and 84 patients (46.4%) constituted the non-MDRO group.

**Table 1:** Demographic and clinical characteristics

	Primary cohort (*n* = 331)	Validation cohort (*n* = 181)
Clinical characteristics		
Age, years	64.00 [52.00, 75.00]	56.00 [43.00, 69.00]
Female, No (%)	125 (37.8)	45 (24.9)
Previous hospitalization, No (%)	105 (31.7)	40 (22.1)
Diabetes, No (%)	80 (24.2)	34 (18.8)
Chronic lung disease, No (%)	32 (9.7)	17 (9.4)
Chronic renal disease, No (%)	34 (10.3)	5 (2.8)
Liver cirrhosis, No (%)	36 (10.9)	6 (3.3)
Bulbar palsy, No (%)	8 (2.4)	7 (3.9)
Immunosuppressive status, No (%)	41 (12.4)	8 (4.4)
MDRO, No (%)	178 (53.8)	97 (53.6)
Organ function status		
Need of vasopressor agents, No (%)	101 (30.5)	55 (30.4)
ALB, g/L	29.50 [25.80, 32.65]	29.00 [25.00, 34.00]
TBil, umol/L	17.30 [9.10, 33.10]	18.40 [11.90, 25.00]
PLT < 100 × 10^9^/L, No (%)	98 (29.9)	33 (18.2)
Cr, umol/L	76.00 [55.00, 135.50]	91.00 [65.00, 124.00]
PaO_2_/FiO_2_, mmHg	224.00 [153.50, 305.50]	233.00 [175.00, 342.00]
Indwelling catheter		
Tracheal intubation / incision, No (%)	218 (65.9)	133 (73.5)
Indwelling urethral catheter, No (%)	308 (93.1)	169 (93.4)
Indwelling central venous catheter, No (%)	289 (87.3)	81 (44.8)
Laboratory indicators		
WBC, 10^9^/L	11.96 [7.69, 17.43]	14.30 [11.70, 18.00]
N%	87.50 [81.40, 91.90]	87.80 [83.90, 91.20]
L%	6.10 [3.50, 10.90]	6.40 [4.50, 10.00]
NLR	14.26 [7.61, 25.49]	13.61 [8.67, 19.80]
PCT, ug/L	1.86 [0.46, 16.93]	0.38 [0.10, 2.46]
CRP, mg/L	67.90 [24.00, 90.00]	98.10 [47.90, 169.50]
Scores		
SOFA	7.00 [4.00, 9.00]	7.00 [5.00, 9.00]
APACHEII	14.00 [11.00, 18.50]	20.00 [14.00, 25.00]
Pitt	2.00 [1.00, 4.50]	4.00 [2.00, 7.00]

The quantitative data are normal distribution, expressed by mean ± standard deviation; The quantitative data were skewed and expressed in the median (25–75% percentile); Qualitative data were expressed in n%; *MDRO* multidrug-resistant organism; *Alb* albumin; *TBil* total bilirubin; *PLT* platelet; *Cr* creatinine; PaO_2_/FiO_2_, oxygenation index; WBC, leucocyte count; N%, neutrophil percentage; L%, lymphocyte percentage; *NLR* neutrophil-to-lymphocyte ratio; *PCT* procalcitionin; CRP, C- reactive protein; SOFA score, Sequential Organ Failure Assessment score; APACHEII score, Acute Physiology and Chronic Health Evaluation (APACHE) II score; Pitt score, Pitt bacteremia score

### Independent predictors of MDRO colonization or infection

Univariate analysis indicated that male sex, higher CRP levels and higher Pitt scores were associated with MDRO colonization or infection (Table 2). Multivariate analysis continued to demonstrate that the above three variables were independent predictors for MDRO colonization or infection, including female sex (odds ratio [OR] 0.56, 95% confidence interval [CI] 0.35–0.89, *p* = 0.014), CRP (OR 1.06, 95% CI 1.02–1.09, *p* = 0.002) and Pitt score (OR 1.13, 95% CI 1.03–1.22, *p* = 0.006) (Table 2).

**Table 2:** Logistic regression analysis of predictive factors for MDRO colonization or infection in ICU patients

	Univariate logistic regression analysis		Multivariate logistic regression analysis	
	OR (95% CI)	*P* value	OR (95% CI)	*P* value
Age, years	1.004 (0.99–1.02)	0.526		
Female	0.59 (0.38–0.92)	0.021	0.56 (0.35–0.89)	0.014
Previous hospitalization	1.22 (0.77–1.95)	0.403		
Diabetes	1.40 (0.84–2.34)	0.201		
Chronic lung disease	1.29 (0.62–2.76)	0.505		
Chronic renal disease	1.44 (0.70–3.05)	0.326		
Liver cirrhosis	0.66 (0.32–1.31)	0.237		
Bulbar palsy	0.51 (0.10–2.10)	0.358		
Immunosuppressive status	1.40 (0.72–2.78)	0.325		
Need of vasopressor agents	1.04 (0.65–1.67)	0.870		
ALB, g/L	1.03 (0.99–1.07)	0.158		
TBil, umol/L	1.00 (0.99–1.003)	0.379		
PLT < 100 × 10^9^/L	1.13 (0.70–1.83)	0.609		
Cr, umol/L	1.00 (0.99–1.002)	0.912		
PaO_2_/FiO_2_, mmHg	1.00 (0.99–1.002)	0.784		
Tracheal intubation / incision	1.44 (0.91–2.29)	0.116		
Indwelling urethral catheter	1.56 (0.67–3.76)	0.308		
Indwelling central venous catheter	1.33 (0.69–2.55)	0.393		
WBC, 10^9^/L	0.99 (0.97–1.02)	0.650		
N%	0.99 (0.97–1.01)	0.442		
L%	1.01 (0.98–1.05)	0.391		
NLR	1.00 (0.99–1.01)	0.879		
PCT, ug/L	1.00 (0.99–1.004)	0.430		
CRP, 10 mg/L	1.05 (1.02–1.09)	0.004	1.06 (1.02–1.09)	0.002
SOFA	0.99 (0.93–1.05)	0.811		
APACHEII	1.01 (0.97–1.05)	0.570		
Pitt	1.13 (1.04–1.23)	0.003	1.13 (1.03–1.22)	0.006

*MDRO* multidrug-resistant organism; *Alb* albumin; *TBil* total bilirubin; *PLT* platelet; *Cr* creatinine; *PaO*_*2*_*/FiO*_*2*_ oxygenation index; *WBC* leucocyte count; N%, neutrophil percentage; L%, lymphocyte percentage; *NLR* neutrophil-to-lymphocyte ratio; *PCT* procalcitionin; *CRP* C- reactive protein; *SOFA score* Sequential Organ Failure Assessment score; *APACHEII score* Acute Physiology and Chronic Health Evaluation (APACHE) II score; Pitt score, Pitt bacteremia score

### The nomogram for predicting the occurrence of MDRO colonization or infection: performance and validation

**Fig. 2:**
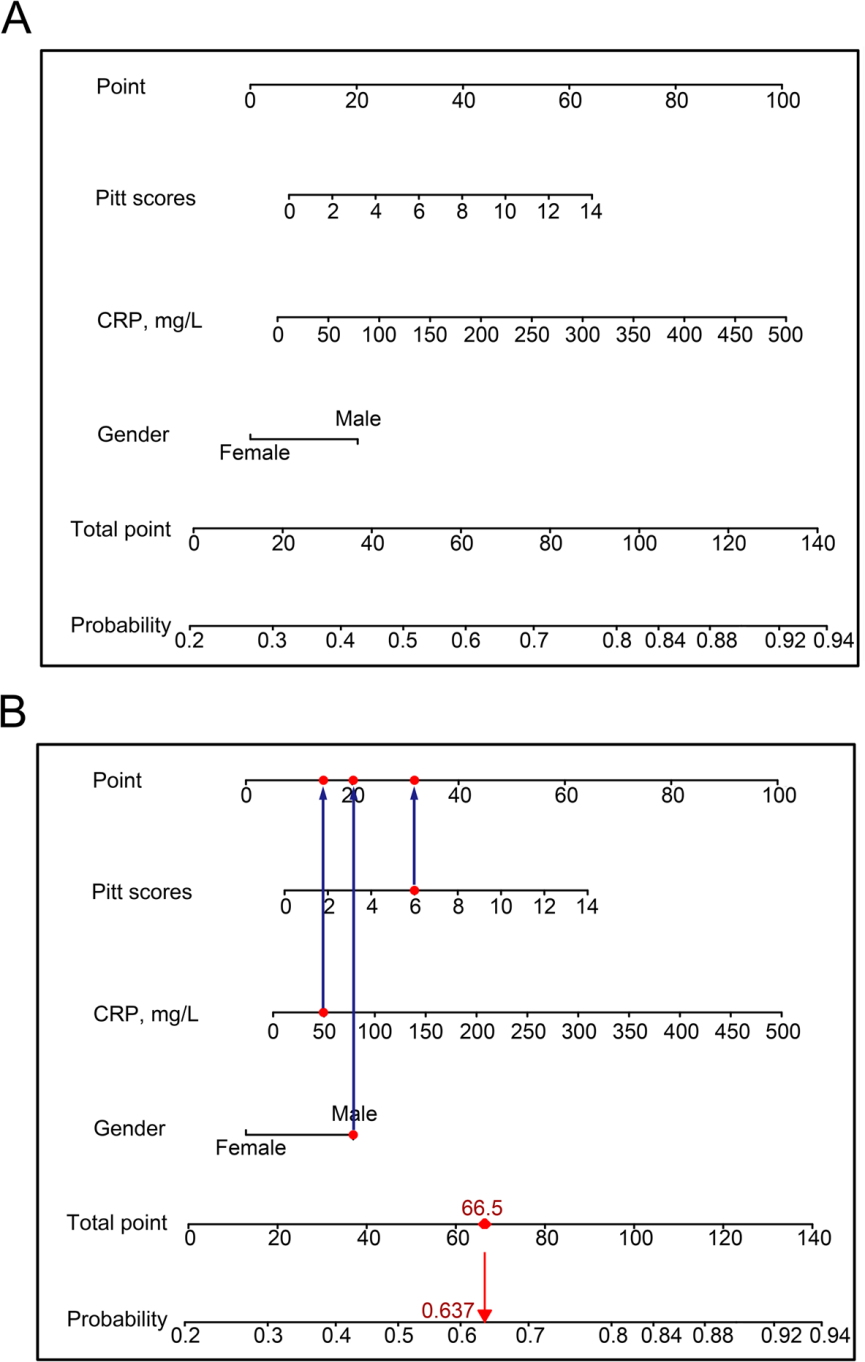
**a** The nomogram to predict the occurrence of MDRO colonization or infection in ICU patients. This nomogram provides a method of calculating the probability of the occurrence of MDRO colonization or infection in the ICU based on a patient’s combination of covariates in the early stages of the ICU stay. To use the nomogram, locate the patient’s gender, and draw a line straight up to the points axis to establish the score associated with that gender. Repeat for the other two covariates. Add the scores for each covariate together, and locate the total score on the total points axis. Draw a line straight down to the linear predictor axis to obtain the probability. **b**. An example of how to use the nomogram to predict the occurrence of MDRO colonization or infection in ICU patients. A male patient has a CRP value of 50 mg/L within 48 h of entering the ICU, and his Pitt score is 6 points. The points for each predictor add up to 66.5. A vertical line is then drawn from 66.5 on the “Total points” line down to the last lines to predict further MDRO colonization or infection (63.7%)

We created a nomogram to predict the probability of the occurrence of MDRO colonization or infection in ICU patients (Fig. 2a). The nomogram integrated all three significant independent predictors determined from the multivariate analysis in the primary cohort. When using the nomogram, the patient’s gender was located, and a line was drawn straight up to the points on the axis to establish the score associated with that gender. This process was repeated for the other two covariates. The scores for each covariate were added, and the total score on the axis depicting the total points was located. A line was drawn straight down to the linear predictor axis to obtain the probability. An example of how to use the nomogram to predict the occurrence of MDRO colonization or infection in ICU patients is presented in Fig. 2b.

**Fig. 3:**
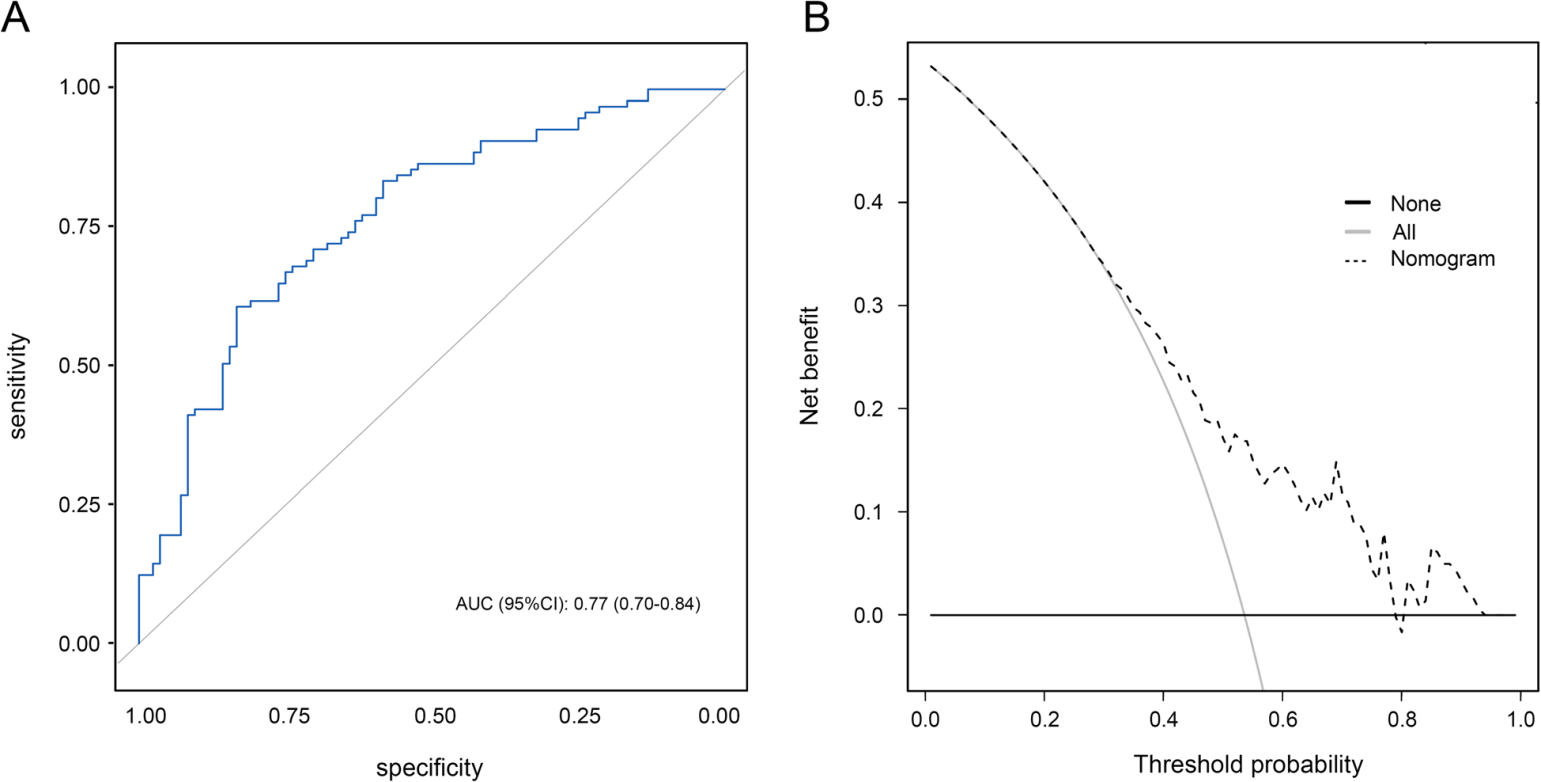
**a**. The receiver operating characteristic curves for the nomogram in the external validation cohort. The AUC for our model was 0.77 (95% CI 0.70–0.84). **b**. Decision curves for the overall incidence of MDRO colonization or infection according to the nomogram in the external validation cohort. Gray line: net benefit of a strategy of predicting all patients; black line: net benefit of predicting no patients; dotted line: net benefit of predicting patients according to the nomogram. The nomogram-based decisions were supported in the range of threshold probabilities of approximately 30–95%

The predictive ability of the nomogram was externally confirmed in the validation cohort. The AUC for the nomogram was 0.77 (95% CI 0.70–0.84), indicating good discrimination (Fig. 3a). Figure 3b illustrates the decision curves for the nomogram. The gray line represents the net benefit of a strategy of predicting all patients. The black line indicates the net benefit of predicting no patients. The dotted line represents the net benefit of predicting patients according to the nomogram. The nomogram-based decisions were supported in the range of threshold probabilities of approximately 30–95%, indicating the clinical usefulness of the nomogram.

### The distribution of MDROs in the primary cohort

In 178 cases of MDROs in the primary cohort, Acinetobacter baumannii (*n* = 62, 30.0%) was the most common among gram-negative bacteria, followed by *Escherichia coli* (*n* = 51, 24.6%); among gram-positive bacteria, the majority of cases involved *Enterococcus faecium* (*n* = 17, 37.8%), followed by methicillin-resistant Staphylococcus aureus (MRSA) (*n* = 9, 20.0%) (Table 3).

**Table 3:** Bacterial species of 178 patients with MDROs in the primary cohort

Species	n	Percentage
Gram-negative bacterium	207	
Acinetobacter baumannii	62	30.0%
*Escherichia coli*	51	24.6%
*Klebsiella pneumoniae*	40	19.3%
*Pseudomonas aeruginosa*	24	11.6%
Stenotrophomonas maltophilia	11	5.3%
*Burkholderia cepacia*	8	3.9%
Meningitis Elizabeth	3	1.4%
*Enterobacter cloacae*	3	1.4%
Citric acid bacteria	2	1.0%
*Haemophilus influenzae*	1	0.5%
*Proteus mirabilis*	1	0.5%
Sphingosporin oligosporium	1	0.5%
Gram-positive bacterium	45	
*Enterococcus faecium*	17	37.8%
MRSA	9	20.0%
Staphylococcus haemolyticus	7	15.6%
Staphylococcus capitatus	4	8.9%
MSSA	4	8.9%
Coagulase negative-	3	6.7%
Staphylococcus		
Streptococcus pneumoniae	1	2.2%

*MDROs* Multiple drug-resistant organisms; MRSA, methicillin-resistant Staphylococcus aureus; *MSSA* methicillin-sensitive Staphylococcus aureus; 2 species of MDROs colonization or infection in 50 cases; 3 species of MDROs colonization or infection in 12 cases; Both gram-positive and gram-negative MDROs colonization or infection in 26 cases

## Discussion

With the widespread use of antibiotics, bacterial drug resistance is becoming increasingly serious. However, progress in treatment has been limited [4]. Rational use of antibiotics is of importance to prevent and control MDRO infections [4, 5, 23].

For empirical treatment decision assistance, we identified independent parameters from data available in the early stage of the ICU stay and constructed a novel nomogram for predicting the occurrence of MDRO colonization or infection.

Our nomogram has several strengths. A remarkable strength is its ease of use. The parameters obtained from the patient status in the early stages of the ICU stay are well defined, easily measured, and routinely available. The construction of our model using the Pitt score, CRP level and gender, which do not require information about the diagnosis and detailed medical history, may present an advantage in the ICU setting [15, 16]. Another advantage of our nomogram is that it was subjected to an independent external validation process and showed good discrimination. In addition, the use of the nomogram has another advantage over previous models. As long as the results of relevant factors are input into the nomogram according to the steps shown in Fig. 2b, the corresponding results are obtained easily. This helps in quantitatively calculating the probability of MDRO colonization or infection.

The nomogram established in the present study incorporated three factors, namely, the male sex, CRP level, and Pitt score. CRP is an inflammatory marker that has been used in clinical practice for decades [24]. Many previous studies have suggested the utility of the CRP level as an outcome predictor in critically ill patients with sepsis [25, 26]. We determined for the first time that the CRP level also has predictive value for MDRO colonization or infection. Pitt scores have been widely used to evaluate the prognosis and infection potential of critically ill patients [27, 28]. To the best of our knowledge, the present study is the first to focus on assessing the Pitt score as an indicator of MDRO colonization or infection. In a study on nosocomial sepsis by Kavitha S et al., a high degree of multidrug resistance was observed among both gram-positive and gram-negative organisms in patients with nosocomial sepsis, and male gender was the independent predictor of mortality [29]. These findings may confirm to some extent the relationship between male gender and MDROs infection. In other words, all the factors revealed as significantly predictive in our study are plausible.

Notably, the nomogram only applies to adult patients in the ICU. Its purpose is to predict the occurrence of MDRO colonization or infection during the ICU stay, excluding MDROs that are detected before entry into the ICU or within the first 48 h in the ICU.

Some limitations must also be recognized when interpreting the results. First, the nomogram was developed based on data obtained retrospectively at a single center, and only patients with microbial culture were included in the study. Further studies are warranted to explore whether this nomogram can be extended to all ICU patients. Second, other valuable predictors may have been ignored in our analysis. Our nomogram might be improved as additional predictive variables are incorporated; it is not a finished product that is perfectly able to predict MDRO colonization or infection in the ICU. Third, our nomogram only predicts the occurrence of MDRO colonization or infection, but does not implicate any causal relationship between gender, CRP, Pitt score and occurrence of MDRO colonization and/or infection.

## Conclusions

Three independent predictors, male sex, higher CRP level and higher Pitt score, were identified in our study. These predictors are readily available during the early ICU stay and can be assembled to construct an easy-to-use nomogram predicting MDRO colonization or infection. Thus, the nomogram is potentially useful for predicting the occurrence of MDRO colonization or infection in ICU patients.

## Data Availability

The datasets used and/or analysed during the current study are available from the corresponding author on reasonable request.

## Abbreviations

MDROs: Multidrug-resistant organisms
ICU: Intensive care unit
ROC: Receiver operating characteristic
AUC: Area under the receiver operating characteristic curve
CRP: C-reactive protein
Pitt scores: Pitt bacteremia scores
NLR: Neutrophil-to-lymphocyte ratio
N: Absolute neutrophil count
L: Absolute lymphocyte count
ALB: Albumin
Cr: Creatinine
TBil: Total bilirubin
PLTs: Platelets
PaO_2_/FiO_2_: Oxygenation index
WBC: Leucocyte count
N%: Neutrophil percentage
L%: Lymphocyte percentage
PCT: Procalcitionin
SOFA: Sequential Organ Failure Assessment score
APACHE II: Acute Physiology and Chronic Health Evaluation II score
AIC: Akaike Information Criterion
OR: Adds ratio
CI: Confidence interval
MRSA: Methicillin-resistant *Staphylococcus aureus*
MSSA: Methicillin-sensitive Staphylococcus aureus
